# A Retrospective Analysis of Incidence and Its Associated Risk Factors of Upper Urinary Tract Recurrence following Radical Cystectomy for Bladder Cancer with Transitional Cell Carcinoma: The Significance of Local Pelvic Recurrence and Positive Lymph Node

**DOI:** 10.1371/journal.pone.0096467

**Published:** 2014-05-05

**Authors:** Sung Han Kim, Hyung-Kook Yang, Jung Hoon Lee, Eun-Sik Lee

**Affiliations:** 1 Department of Urology, National Cancer Center, Goyang, Gyanggi, Korea; 2 Department of Epidemiology and Statistics and Cancer Policy Branch of the National Cancer Control Research Institute, National Cancer Center, Goyang, Gyanggi, Korea; 3 Department of Urology, Seoul National University Hospital, Seoul, Seoul, Korea; Health Canada and University of Ottawa, Canada

## Abstract

**Objective:**

The aim of this study is to examine the incidence and risk factors of upper urinary tract recurrence (UUTR) following radical cystectomy (RC) in bladder cancer and to evaluate its relationship with neobladder (Neo) or ileal conduit (IC).

**Materials and Methods:**

All clinicopathologic parameters and perioperative parameters of 311 patients who underwent RC with either Neo or IC by a single surgeon from 1999 to 2012 were retrospectively included in this study. Patients with a history of renal surgery, concomitant UUTR, or a histopathology of non-transitional cell carcinoma were excluded. For statistical analyses of predictive risk factors of UUTR, a multivariate analysis was performed with known risk factors of UUTR, including type of urinary diversion with significance defined as *P* < 0.05.

**Results:**

During the median follow-up period of 53 months, 143 (46.0%) IC and 168 (54.0%) Neo were performed, resulting in 11 (3.5%) cases of UUTR (Neo 7 and IC 4) after RC and all patients then underwent nephroureterectomy. No significant differences in incidence and overall survival in UUTR were observed according different types of urinary diversion (p = 483), and the prognosis for survival of Neo was insignificantly better than that of IC (5-year overall survival 78% vs 74%, respectively, p>0.05). Higher number of positive lymph nodes (HR 9.03) and the presence of pelvic local recurrence (HR 7286.08) were significant predictive factors of UUTR (p<0.05).

**Conclusion:**

This study reports a UUTR rate of 3.5%, and positive lymph nodes and presence of local recurrence at the pelvis as important risk factors. No significant differences in incidence and survival were observed between Neo and IC.

## Introduction

Approximately 20–30% of cases of bladder cancer (BC) are reportedly found at muscle-invasive status at initial presentation and even 20–50% of early detected superficial BC also progressed to invasive, with a loco-regional recurrence rate of 5–15% during follow-ups [1], [2]. For muscle-invasive or frequently recurred BC, radical cystectomy (RC) with urinary diversion of either neobladder or ileal conduit is a standard treatment, and upper urinary tract recurrence (UUTR) is another important issue during postoperative follow-ups. However, because incidence of UUTR following RC for BC is relatively rare, ranging between 0.74% and 6.4%, and prognosis is poor, with median survival within 24 months after development, and with late occurrence until nine years after RC, the characteristics and clinical course of UUTR have not yet been fully defined and the different follow-up strategy of evaluating upper urinary tract has still been discussed [3]–[10]. Various risk factors for UUTR have been reported, such as age, nuclear grade, stage, multifocality, histology, a positive margin of urethra or ureter, lymph nodal positivity, and the presence of carcinoma in situ (CIS) [3], [6], [7], [11]–[14]. Therefore, prediction of an increased likelihood of UUTR and its risk factors is important in order to define the strategy for monitoring during follow-up [9], [10], [12].

In this study, we reviewed our computerized RC Database Registry in order to examine the incidence and clinical course of UUTR after RC and to determine its predictive risk factors. In addition, the relationship between the incidence of UUTR and the different types of urinary diversion of RC was also evaluated.

## Materials and Methods

Of the 366 RC patients treated for primary BC since 1992, the records of 311 patients who underwent RC with 168 orthotopic (neobladder) or 143 non-orthotopic (ileal conduit) urinary diversion by a single surgeon (ESL) between April 1999 and December 2012 were reviewed. Patients were censored at last follow up or date of death due to BC and other causes according to the cancer database in the Korean National Insurance Health Statistics, where all cancer patients in Korea should be automatically registered and followed up until death. No deaths or UUTR were detected among patients who were lost to follow-up or transferred to another hospital because of the accessibility of distance. Minimum follow up period for living patients was one year. Indications for RC included muscle invasive BC, CIS refractory to Bacillus Calmette-Guerin intravesical therapy, recurrent multifocal high grade superficial BC refractory to transurethral resection, and large superficial papillary BC with severe comorbidities without muscle invasive at initial transurethral section, but not enough to perform multiple transurethral operations. We excluded patients who underwent palliative cystectomy for control of bleeding and pain relief, who had a previous history of renal surgery before RC as well as concomitant UUTR at RC, who had no history of postoperative follow-up at our institution, or who had non-transitional cell carcinoma on final pathologic specimen of bladder at RC.

UUTR was defined as any abnormal findings of occurred and proven cancer recurrence in radiography, endoscopy, or pathology along the upper urinary tract [7]. Cancer recurrence and pathological staging, including UUTR and RC specimens, were staged and graded according to the TNM classification (TNM), the World Health Organization system (WHO), and American Joint Committee on Cancer criteria (AJCC) [15]–[17]. Nephroureterectomy (NU) was also graded, pathologically staged with histology. All preoperative and postoperative pathological studies were reviewed by pathologists. In addition, the mode of clinical manifestation and method of diagnosis, time to recurrence, type of therapy, and clinical outcomes were collected retrospectively by chart reviews.

### Preoperative Evaluation

Preoperative evaluation included cystoscopy, washing cytology, transurethral resection of bladder tumor and/or bladder biopsy with anesthesia, upper urinary tract (UUT) imaging with excretory urography (EXU) or intravenous pyelography (IVP) and/or enhanced abdominal computed tomography (CT), and chest X-ray. Some patients underwent further metastatic evaluation, including whole-body bone scan (WBBS), chest CT, and/or positron emission tomography-CT (PET-CT), as clinically indicated.

### Additional treatment

Neoadjuvant or adjuvant chemotherapy was administered based on the decision of the surgeon. The indication for neoadjuvant chemotherapy was those patients suspicious for whether single nodal positive or microscopically extravesical extended states on imaging studies and cytoscopic findings with endoscopically resected specimens. The indication for adjuvant chemotherapy was routinely recommended for all node-positive advanced or newly developed metastatic patients at our institute, except for those who were medically intolerant or who refused this treatment. Most patients with neoadjuvant chemotherapy received two to four cycles of a combination of gemcitabine and carboplatin/cisplatin (GC/GP) according to the functional status of their kidney. Most patients with adjuvant chemotherapy received a combination of GC/GP or methotrexate, vinblastine, doxorubicin, and cisplatin (MVAC) until no response. The cycles and combination of chemotherapeutic agents for neoadjuvant and adjuvant chemotherapy were performed according to the extent of coverage by the national insurance policy, tumor response, and general performance status including underlying disease such as chronic kidney disease with an estimated glomerular filtration rate less than 60 mL/min/1.72 m^2^.

### Operative procedure

RC was completed, including routine serial intraoperative frozen section examination of the ureters above the crossing iliac vessels and/or the urethra until the resection was clear. These results determined the need for subsequent resection of the proximal ureter, NU, and urethrectomy. Redundant ureter excised at the time of ureteroenteric anastomosis constituted the final specimen. Lymph node dissection and its extent of dissection (none, limited, standard, and extended lymph node dissection [LND]) were completed according to surgeon decision that any nodal involvements during preoperative imaging work ups or intraoperative suspicious lymph nodal enlargements, or clinical tumor stages were indicated for performance of LND. The extent of LND was determined according to previous reports [18], [19].

### Postoperative follow-up protocol

The follow-up schedule was determined according to the pathology of the RC specimen. Patients with pT2 or lower without nodal involvement underwent follow-up examinations every 3–6 months for two years after RC, and then every 6–12 months. Patients with pT3-4 or nodal positive underwent follow-up examinations every three months after RC. Regular follow-up examinations included physical examination, blood chemistry studies, complete blood count, urinary analysis and cytology, chest X-ray and EXU or IVP or enhanced abdominal CT. Further imaging, including retrograde ileography, was performed if recurrence was suspected based on unexplained symptoms or clinical concern. In the absence of recurrence for five years after RC, the interval between examinations was extended based on the decision of the surgeon in consideration of tumor pathology with intraoperative disease states and clinical severity. Patients diagnosed with UUTR underwent metastatic evaluation, including abdominal CT, chest X-ray, and WBBS or PET.

### Statistical measures

For the statistical analysis, all clinicopathologic parameters and perioperative parameters were considered. Statistical analyses were performed by an experienced medical statistician (HK.Y) using independent t-test and Mann-Whitney t-tests for numerical variables and Chi-square and Fisher’s exact tests for categorical variables to compare the UUTR incidences between different urinary diversions of RC. Multivariate model was performed by logistic regression analysis among variables found to be both clinically and statistically significant in univariate analysis.

To define clinical prognosis of survival and recurrence with their associated risk factors for UUTR, the Kaplan–Meier method, log–rank test, and Cox proportional hazards model were applied using SPSS 18.0 (SPSS Inc., Chicago, USA). Two-sided statistical analyses were performed and significance was defined as *P* < 0.05.

### Ethics statement

An ethics committee and an institutional review board (IRB) of Seoul National University Hospital approved computerized RC Database Registry was used to report clinical outcomes of UUTR after RC for primary BC (IRB No. H1208-081-422). All the patients participated in his study did provide verbal informed consent and the ethics committees/IRB approved this consent procedure and the exemption of written informed consent.

## Results

The median age of 311 patients was 64.0 (46–86) years old with a median follow-up period of 53.0 (13-207) months. Male (88.7%) was more predominant than female (11.3%). For types of urinary diversion, 143 IC (46.0%) and 168 Neo (54.0%) were performed. Intravesical, neoadjuvant, and adjuvant chemotherapies were administered for 64 (20.5%) (4 GC and 60 GP based regimen) and 83 (26.7%) patients (76 GC and 7 MVAC based regimen), respectively. LND was performed only in 74.9% at RC. However, all 11 UUTR cases underwent LND at RC. According to pathologic stage, organ-confined disease (pT0-2N0, Tis) was found in 161 patients (51.8%), non-organ-confined disease (pT2-4Nx) in 140 (48.2%), including pT2N+ stage in 20 (6.4%), and other positive lymph nodal metastasis in 21 (6.8%) (Table 1). No significant differences in incidence and overall survival in UUTR were observed according different types of urinary diversion (p = 483), and the prognosis for survival of Neo was insignificantly better than that of IC (5-year overall survival 78% vs 74%, respectively, p>0.05; not shown in tables).

**Table 1:** Patients’ characteristics (n = 311).

	N	%
Median age	64	(46–86)
Male/Female (n,%)	276/35	88.7/11.3
Hypertension/diabetes (n,%)	89/37	28.6/11.9
American society of Anestheology Score (n,%)		
Grade 1	157	50.4
Grade 2	143	46.1
Grade 3	11	3.5
Radical Cystectomy with Orthotopic neobladder (n,%)	168	54.0
with Non-orthotopic ileal conduit	143	46.0
Intravesical chemotherapy (n,%)	45	14.5
Neoadjuvant chemotherapy (n,%)	64	20.5
Extent of LN dissection None (n,%)	78	25.1
Limited	68	21.9
Standard	158	50.7
Extended	7	2.3
Resection margin positivity (n,%)	19	6.1
Ureter/Urethra	6/13	31.6/68.4
No. patients according to pathological tumor stage (n,%)		
pT0, Ta	46	14.8
pTis	29	9.3
pT1	51	16.4
pT2	65	20.9
pT3	84	27.0
pT4	36	11.6
No. patients according to pathological nodal positive (n,%)	41	13.2
Nuclear grade (n,%) Low	34	10.9
Moderate	171	54.9
High	106	34.2
Perineural invasion (n,%)	40	12.9
Lymphovascular invasion (n,%)	87	28.0
Adjuvant chemotherapy (n,%)	84	27.0
Local recurrence (n,%)	39	12.5
Distant metastasis (n,%)	79	25.4
Median follow-up period	53	(13–207)
Survival (n,%)	184	59.2
Median survival time (mo)	46.0	(2–208)

A total of 11 patients (3.5%) from seven Neo and four IC developed UUTR and all patients then underwent NU with only two patients with LND at suspicious peri-hilum and peri-aorta and with only five patients receiving further adjuvant chemotherapy (Table 2). The median time to UUTR after RC was 26 months (not shown in tables). Pathological examination of UUTR patients showed that only two patients had organ-confined disease (pT3aN0) on RC specimens and the remaining nine (81.8%) patients had non-organ-confined disease with five (45.5%) patients with nodal positivity. Local recurrence at the pelvis was observed in six patients, including two urethras, whereas no positive resection margin of ureters and urethra was observed on RC specimens of all 11 patients (Table 2).

**Table 2:** The demographics of 11 patients with UUTR.

Age (yrs)	Diversion type	Pathology	Neoadj and IV CTx	UUTR (mo)	Recur site	Local recur site	Treatment For UUTR	Pathology of UUTR	UUTR surgery to last F/U	Status
51	Neo	TCC, G2, pT3bN1	None	72	Distal ureter		Surgery and Adjuvant CTx	TCC, pT2N1	13	DOD
58	Neo	TCC, G2, pT3bN2	IV CTx	19	Distal ureter		Surgery and Adjuvant CTx	TCC, pT1Nx	4	DOD
58	IC	Papil, G1, pT3bN0	IV CTx	29	Distal ureter	Rectum	Surgery	Pap, T2N1	5	DOD
64	IC	TCC,G3, pT2N2	None	42	Renal Pelvis	Pelvis	Surgery and Adjuvant CTx	CIS, pT1Nx	31	LWD
65	Neo	Pap, G3, pT3bN0	IV CTx	29	Proximal ureter	Pelvis	Surgery	TCC, TisNx	10	DOD
66	Neo	TCC, G3, pT3bN0	Neoadj CTx	19	Proximal ureter	Common iliac LN	Surgery and Adjuvant CTx	TCC, T1Nx	2	DOD
67	Neo	TCC, G3, pT3aN0	None	45	Proximal ureter		Surgery and Adjuvant CTx	TCC, pT1Nx	4	LWD
68	Neo	TCC, G3, pT3bN0	None	14	Distal ureter		Surgery and Adjuvant CTx	TCC, pT2N0	17	LWD
71	Neo	Pap, G1, pT1N2	IV CTx	18	Renal pelvis	Urethra	Surgery and Adjuvant CTx	Pap, pT2Nx	23	LWD
75	IC	TCC, G3, pT4bN2	IV CTx	26	Distal ureter	Urethra	Surgery	Pap, TaNx	6	DOD
83	IC	TCC, G3, pT3aN0	None	17	Distal ureter		Surgery	TCC, pT1Nx	5	LWD

IV, intravescial chemotherapy; CTx, chemotherapy; F/U; follow-up, Neo, neobladder; IC, ileal conduit; DOD, died of the disease; LWD, lived with the disease; TCC, transitional cell carcinoma; Papil, papillary TCC;

In comparison of the 11 patients of the UUTR group with the remaining patients (n = 300, non-UUTR group), besides a significantly poorer general condition referred from the ASA score, higher rate of intravesical instillation of agents before RC, and postoperative local recurrence at pelvis, and shorter duration of follow-up in the UUTR group, no significant differences in clinicopathologic, and perioperative parameters were observed between the two groups (p<0.05, Table 3). In comparison of median survival between the two groups, the UUTR group had insignificantly poorer survival of approximately nine months than the other group (median survival 65.9 vs. 76.9 months, p = 0.383, respectively; Table 3). In assessment of previously known clinicopathological factors from other studies and our significant univariate parameters in prediction of the predictive risk factors of UUTR after RC, the presence of local recurrence at the pelvis (HR 7286.1, CI 3.50-122760.70) and number of positive lymph nodes (HR 9.03, CI 1.22-66.79) were independently significant (p<0.05, Table 4).

**Table 3:** Comparison between UUTR and no-UUTR.

	UUTR (n = 11)	No-UUTR (n = 300)	p-value
Gender Male/Female (n,%)	10 (90.9)/1 (9.1)	266 (88.7)/34 (11.3)	0.605
Age (yr)	65.8±8.5	62.7±10.3	0.314
BMI (kg/m^2^)	24.9±3.8	23.8±3.2	0.283
Hypertension/Diabetes (n,%)	3 (27.3)/1 (9.1)	86 (28.9)/36 (12.1)	>0.500
IV CTx instillation (n,%)	5 (45.5)	40 (13.3)	0.002
Neoadjuvant CTx. (n,%)	1 (9.1)	63 (21.0)	0.730
Adjuvant CTx (n,%)	7 (63.6)	77 (25.7)	0.015
ASA Grade 1 (n,%)	6 (54.5)	151 (50.3)	0.039
Grade 2	3 (27.3)	140 (46.7)	
Grade 3	2 (18.2)	9 (3.0)	
Hospital stay (day)	21.4±11.4	21.4±15.9	0.998
Total operative time (min)	402.7±159.9	379.6±105.6	0.486
Neobladder/ Ileal conduit (n,%)	7/4 (63.6/36.4)	161/139 (53.7/46.3)	0.120
Pathologic T0 or Ta or Tis (n,%)	0	75 (25.0)	0.282
T1	1 (9.1)	49 (16.3)	
T2	1 (9.1)	64 (21.4)	
T3	8 (72.7)	77 (25.6)	
T4	1 (9.1)	35 (11.7)	
Pathologic node positivity (n,%)	5 (45.5)	38 (12.7)	0.182
Nuclear grade low (n,%)	2 (18.2)	32 (10.7)	0.354
moderate	2 (18.2)	169 (56.3)	
high	7 (63.6)	99 (33.0)	
Resection margin positivity (n,%)	0	19 (6.3)	<0.001
Perineural invasion (n,%)	4 (36.4)	36 (12.0)	0.130
Lymphovascular invasion (n,%)	3 (27.3)	84 (28.0)	0.753
Extent of LN dissection (n,%)			0.320
None	0	78 (26.0)	
Limited	1 (9.1)	67 (22.3)	
Standard	9 (81.8)	149 (49.7)	
Extended	1 (9.1)	6 (2.0)	
Local recurrence (n,%)	6 (65.6)	33 (3.7)	<0.001
Distant metastasis (n,%)	2 (18.2)	77 (25.7)	0.734
Follow-up duration (mo)	50.8±19.6	63.8±42.9	0.049
Survival (n,%)	5 (45.5)	179 (59.7)	0.026
Median survival time (mo)	65.9	76.9	0.383

BMI, body mass index; ASA, American Society of Anesthesiologists; IV, intravesical; CTx, chemotherapy; Tis, T stage with carcinoma in situ; Significant as p<0.05.

**Table 4:** Predictive risk factor of UUTR in Poisson regression analysis.

	Hazard ratio	p-value	95% Confidence Interval
Hospital stay	1.00	0.99	0.81–1.23
ASA score	0.97	0.06	0.94–1.00
Type of Urinary diversion (Neo vs IC)	0.00	0.07	0.00–1.79
Positive resection margin	5.62	0.48	0.05–688.53
Lymph nodal dissection	0.42	0.64	0.01–16.17
Positive lymph node	9.03	0.03	1.22–66.79
Adjuvant chemotherapy	5.07	0.46	0.07–372.36
Pelvic local recurrence	7286.08	0.02	3.50–122760.70
Distant metastasis	18.19	0.16	0.33–1012.56
Pathologic T stage	2.20	0.29	0.51–9.55
Pathologic N stage	0.07	0.38	0.00–27.46

## Discussion

For decades, the schedule and methods for surveillance of UUT after RC have been discussed because of its characteristics of low prevalence (0.7–7.4%) [3], [7], [8], [12], [20]–[22], late diagnosis after postoperative 25–40 months [6], [7], [23], poor prognosis with a median survival of 10–20 months from diagnosis [6], [23], difficulty in detection of abnormal UUT findings in the changed abdomino-pelvic anatomy with diverted intestinal urinary tract of urinary diversions, and contaminated urine specimen from UUT with many degenerating desquamated intestinal epithelial cells to decrease the sensitivity of urine cytology. Many previous studies have attempted to identify several predictive risk factors of UUTR, however, their predictive values varied from those reported in other studies to remain controversial due to different characteristics of the enrolled patients [3], [24]. For these limitations, the UUTR was often diagnosed late in a symptomatically advanced state of approximately 30–64%, resulting in poor prognosis [6]. Therefore, an international consensus regarding BC recommended the UUT follow-up regimen with close and thorough examination every six and 12 months with great suspicion [5], [9], [13], [21]. Similar to previous studies, this study included 11 (3.5%) UUTRs diagnosed at median postoperative 26 months after RC, with a median survival of 13 months and 1- and 2- year UUTR-specific survival of 45.5% and 9.1%. In this study, four (36.4%) UUTR patients were diagnosed after symptoms had developed; two cases of gross hematuria, two cases of fever with flank pain. In another case (9.1%), an asymptomatic patient was diagnosed with UUTR after hydronephrosis on a routine CT work-up, and two other (18.2%) patients on abnormal urine cytology.

According to the urothelial characteristics, BC is known as an urothelial cancer affecting the entire urothelium with the risk for development of recurrence from the renal pelvis to the urethra during monitorization [6], [12], [25]. UUTR is considered to be secondary change to the multifocal metachronous nature of these tumors caused

by both panurothelial field damage by carcinogen to independently transform the epithelial cells at different sites from the renal pelvis to the urethra covered with urothelium, and intraluminal seeding and implantation of tumor cells derived from an initial clone [26], [27]. Up to 57% of patients who undergo RC have unsuspected involvement of cancer at the ureter [28], [29] and other sites at RC, despite a localized disease state of BC, because BC not only invades directly to adjacent organs through the muscle layer of the bladder, but also spreads to farther organs via vascular and lymphatic systems [30]. Therefore, the incidence of UUTR was not related to either the type of urinary diversion or to the quality of surgery, including operative skills, but to the efforts of experienced clinicians in detection of UUTR [3], [8], [24]. Some reports have explained the diverse different rates of UUTR from different institutions in that the size of the hospital, the experiences of clinicians, and the thorough and close monitoring schedule after RC were important in detection of UUTR [8].

To overcome the low early detection rate of recurrence in BC and UUTR with current diagnostic modalities, some recent studies have shown the clinical efficacy of some new molecular and epigenetic biomarkers, and immunohistochemical markers in urine and tissue samples from bladder and UUT. The current imaging modalities of UUT monitorization, such as CT, had a burden of cost and radiologic exposure. [3], [6], [31]. In particular, urine cytology has been known as a potent cost effective tool for screening of BC with a sensitivity of 75–84.6% and specificity of 78–100% for detection of recurrence. However, its results varied based on intrpretation, which is dependent to some extent on the experience and ability of cytopathologists and on the proportion of the primary detection rate lower than 27% in all UUTR after RC [31]. Another study showed that the different types of urinary diversion also affected the low detection rate of recurrence (12.3% of UUTR in IC and 10.5% in Neo) [31]. In our series, only two (18.2%) IC patients were diagnosed with UUTR after abnormalities in urine cytology. Therefore, the molecular biomarkers, such as Ki-67, p53, and Rb; epigenetic markers; and urine markers such as NMP22, might be new breakthroughs in better detection of tumor recurrence of UUT after RC in combination with current diagnostic modalities [6], [32].

In general, incidence of UUTR showed close correlation with the time of follow-up after RC. Most cases of UUTR developed within 25–40 months after RC, with an overall cumulative incidence of 4% at the third year and 7% at the fifth year [6], [7], and some cases developed much later, until 10–15 years [12], [13], [21]. In this study, the median follow-up period was 53 months (13–207 months), which was not quite a long enough period of time for follow-up of UUTR in reference to 10–15 years. However, it might be cautiously said that this study may be representative for RC with an acceptable time of follow-up of UUT, because most of our RC patients (approximately 80%) had completed their follow-up with more than postoperative 36–44 months, and most previous reports also showed a median time of follow-up period of between 30–50 months [3], [6], [8], [23]. Therefore, with our median follow-up period, it was worth discussing the natural histories of 11 UUTRs, although conduct of further studies with large numbers of subjects with further long-term follow-ups would be necessary in order to obtain more clinical significance for comparison with other studies.

In this series, 25.1% of patients did not undergo pelvic LN dissection at RC to be criticized for their representation of RC patients. The reason for not performing pelvic LND for these patients was first that most of them were early staged BC (To, Ta, and T1) with a superficial low-graded papillary tumor without muscle invasiveness confirmed by transurethral resection. Those patients were clear in LN on imaging studies and intraoperatively without palpable LN enlargement. Papillary tumor was a morphological characteristic of BC limited to the mucosa with a benign nature of growing intravesically, different from a solid mass [32]. However, for the papillary tumor, it was necessary to first confirm its muscle invasiveness transurethrally, because some of them had a pathogenically invasive nature. Second, the superficial noninvasive BC (T0 and Ta, n = 46, 11.8%) almost filled in the entire bladder lumen, which meant an indirect parameter for tumors too largely broad and/or too multifocal to be managed transurethrally. This frequently recurrent multifocal superficial tumorigenic nature refractory to repeat transurethral resection has been suggested as one of the recommended indications for RC [10], [24]. And, among patients with these tumors, those with old age greater than 70 years who had multiple underlying comorbidities might have suffered from postoperative morbidity after aggressive LND during RC operations of long duration and their recovery may have been delayed. Therefore, in these cases, the patients did not undergo LND at RC. However, despite the numbers of patients, this study included significantly representative numbers of RC and their UUTRs to be discussed.

This study included a large proportion of RC patients with advanced stage (n = 72, 23.2%) (T3 27.0%, T4 11.6%, and N (+) 13.2%). Especially considering the 11 UUTRs, all Neo patients had pathological stage III. This high proportion of enrolled patients with advanced stage might be attributed one of the ethnic and geographic characteristics of Asian patients with BC. Previous international studies have also shown the existence of disparities among ethnicities and geographic locations, including delays in presentation/diagnosis, inherent biological behavior of the tumor, socioeconomic status, lack of adequate health care access, and differences in exposure to environmental risk factors such as smoking [33]–[35]. The study showed a high prevalence of non-organ confined and high grade disease in Japanese and Taiwanese people and poorer cancer specific and recurrence free survivals were noted [34], [36], similar to our study. Therefore, this study was clinically significant in showing the disease characteristics of BC and UUTR in Korean patients in relation to their ethnic differences from Western peoples, because few Korean studies have reported on UUTR after RC [30], [37].

Among the enrolled patients, four patients received carboplatin-based neoadjuvant chemotherapy. Four patients were suspicious for advanced state with single or multiple small positive pelvic LNs at preoperative imaging evaluation. The cisplatin-based combination regimen has been recommended for its clinical efficacy as both neoadjuvant and adjuvant chemotherapy for invasive or advanced BC, and carboplatin proved less effective with deceased efficacy [38], [39]. However, in our series, carboplatin based chemotherapy was used because of cisplatin-induced nephrotoxicity, coverage of national insurance, and the anti-tumor efficacy of carboplatin is not inferior to that of cisplatin-based chemotherapy. First, as one patient had baseline chronic kidney disease, the less nephrotoxic carboplatin was used. Second, the guideline of Korean National Insurance covered cisplatin only once, in either neoadjuvant or adjuvant chemotherapies, so that two patients who were suspicious for locally advanced state of BC might need adjuvant chemotherapy after RC. The final reason was that carboplatin based regimen preserved a tumor effect identical to that of the cisplatin based regimen, such as MVAC and GP, and that the maximum response might be obtained immediately after the second course because of the short antitumor effect of carboplatin to facilitate the shorter interval between diagnosis and RC [40], [41].

This study did not require a discussion of the quality of surgery, including interpersonal differences of operative skills and distorted proportions of urinary diversion due to its equivalent proportion of Neo and IC (46.0% of IC vs. 54.0% of Neo) compared with other studies [3], [7], [8], [12], [13]. Despite the known dis-relationship of UUTR with the quality of surgery, the resection margin of ureter, urethra, and perivesical tissues and pelvic local recurrence closely related to the UUTR were mostly affected by the operator’s skills during the complex, and time consuming RC procedure, especially in Neo formation [11], [30], [42], [43]. In this study, despite the clear resection margin on frozen section, the final pathologies turned positive margins in 6.1% of cases, similar to other previous reports (false positive and negative 2–6%) [44], [45]. All 11 UUTRs had clear resection margins on final pathology, although six patients later developed UUTR at the distal ureter. This distal UUTR might be explained by a panurothelial field effect in urothelial carcinoma. According to another explanation from Lee et al, intraoperative frozen resection was not an accurate enough analysis to believe the clear UUT after resection of the ureter at RC, because the final pathology indicated that the distal ureter was positive for intramural or juxtavesical ureter involvement and incidence of UUTR development was higher for these conditions [30].

This study analyzed the predictive risk factors for UUTR with most of the previously suggested risk factors from other studies [3], [7], [8], [12], [13], such as a positive ureteral/urethral/perivesical margin, presence of CIS, pathological stage, nuclear grade, local recurrence, lymph nodal positivity and recurrent tumors within urinary diversion, including uretroenteric anastomosis (Table 2) [7], [8], [12], [15]. Local recurrence at the pelvis (HR 7286.08, CI 3.5-122760.7, p = 0.02) and positive LN (HR 9.03, CI 1.22-66.79, p = 0.03) were significant risk factors in this study, which have already been proven in other studies. Other well known risk factors, including stage, ureteral and urethral margin involvement, CIS, and grades, did not reach statistical significance and differed from those of other studies [6], [20], [46] because of small numbers of UUTR cases, which decreased the statistical power. However, our results were similar to those of Balaji et al [22] in that only locally advanced stage was a significant factor without the significance of margins, type of urinary diversion, gender, CIS, and nuclear grade. In this study, 12.5% of local recurrence occurred at a mean time of less than 16.7 (±20.5) months, in accordance with results of previous studies [7], [8], [12], [15]. Three out of five living patients had T3 staged with nodal negative and without local recurrence at the pelvis. The other two patients had staged T1 and T2 despite being nodal positive on the RC specimen and only one local recurrence at the pelvis was noted. Clinically, results of this study indicated that risk of development of UUTR was higher with advanced staged tumors when local recurrence at the pelvis was found during the follow-up period and an increase in the number of positive LN was observed on the final pathology (Table 2). Therefore, urologists should perform a much more careful examination of the UUT with suspicion in these high risk patients.

The type of urinary diversion did not influence the incidence of UUTR. The two different types of urinary diversion were compared in this study under the assumption that incidence of UUTR might be higher with Neo, with poorer prognosis than for IC because Neo has a longer time of mucosal contact from malignant cells in urine and a higher rate of reflux to the upper urinary tract than IC [6], [8], [12], [13], [23]. A study by Yang et al reported a long-term effect of UUT change in both functional deterioration and anatomical distortion because of the refluxing and infection related problem [47]. However, this study showed no significant differences of UUTR, similar to other reports [3], [22]. This might be suggested that the refluxing urine and longer urinary stasis in the Neo do not increase the risk of UUTR. The frequently timed urinary excretion, peristaltic movement of urine from the UUT, and functional anti-refluxing mechanism of Neo prevent from postoperative reflux of urine resulting in no differences in prevalence of UUTR compared to IC [48]. In addition, the prognosis was opposite to our assumption that IC had insignificantly poorer survival because their preoperative baseline performance states and clinical stages were worse not to qualify for Neo (p>0.05, not shown in tables).

The current study has some limitations. A prospective long-term multicenter study with a large number of patients should be conducted in the future for evaluation of suggested risk factors and longitudinal risk assessment of UUTR for evaluation of the potential implications for long-term surveillance. Second, due to its retrospective nature, this study did not include data on biomarkers, therefore, future study would consider the number of risk factors [8], and biomolecular and immunohistochemical markers [5], [49], [50] with the rate of UUTR. Third, because not all patients underwent LN dissection, this study had a limitation of representation for RC. Finally, the number of UUTR was too small, which weakened the statistical power of other well known risk factors. Despite these limitations, we believe that the current results are clinically informative and will be helpful in strategizing the follow-up plan for patients at risk for UUTR after RC.

In conclusion, this study reports on clinical prognosis and incidence of UUTR after RC with its significant risk factors that the incidence of UUTR was 3.5%, and that the presence of local recurrence at the pelvis and positive LNs were important independent risk factors requiring more careful consideration during follow-ups.
